# Population Dynamics Constrain the Cooperative Evolution of Cross-Feeding

**DOI:** 10.1371/journal.pone.0004115

**Published:** 2009-01-05

**Authors:** James J. Bull, William R. Harcombe

**Affiliations:** 1 Integrative Biology, University of Texas, Austin, Texas, United States of America; 2 Institute for Cellular and Molecular Biology, University of Texas, Austin, Texas, United States of America; 3 Center for Computational Biology and Bioinformatics, University of Texas, Austin, Texas, United States of America; Oxford University, United Kingdom

## Abstract

Cross-feeding is the exchange of nutrients among species of microbes. It has two potential evolutionary origins, one as an exchange of metabolic wastes or byproducts among species, the other as a form of cooperation known as reciprocal altruism. This paper explores the conditions favoring the origin of cooperative cross-feeding between two species. There is an extensive literature on the evolution of cooperation, and some of the requirements for the evolution of cooperative cross-feeding follow from this prior work–specifically the requirement that interactions be limited to small groups of individuals, such as colonies in a spatially structured environment. Evolution of cooperative cross-feeding by a species also requires that cross-feeding from the partner species already exists, so that the cooperating mutant will automatically be reciprocated for its actions. Beyond these considerations, some unintuitive dynamical constraints apply. In particular, the benefit of cooperative cross-feeding applies only in the range of intermediate cell densities. At low density, resource concentrations are too low to offset the cost of cooperation. At high density, resources shared by both species become limiting, and the two species become competitors. These considerations suggest that the evolution of cooperative cross-feeding in nature may be more challenging than for other types of cooperation. However, the principles identified here may enable the experimental evolution of cross-feeding, as born out by a recent study.

## Introduction

Microbial communities abound with various forms of cross-feeding [1], [2]. Most generally, cross-feeding involves the metabolic output of one species being used as a nutrient or energy source by another species. In some cases, however, the cross-feeding is two-way and obligate, as in the association between methanogens and ethanol fermenters [3]. Additionally, recent discoveries suggest two-way cross-feeding in the methane consuming association between anaerobic, methane-oxidizing archaea and sulfur-reducing bacteria [1], [4], and in the phototrophic association between green-sulfur bacteria and the β-proteobacteria they encase epibiotically [5]. Consortia of microbes are used in varied industrial purposes, such as food processing, waste degradation, and separating base metals from mineral ore [6]. A consortium of multiple species often has the advantage of performing a task that no single species can perform, and cross-feeding may sometimes be an essential component of such consortia.

Cross-feeding poses several challenges to the biologist, such as the relationship between cross-feeding and community dynamics as well as the evolutionary origin and maintenance of cross-feeding itself. In many examples, cross-feeding can be interpreted merely as one species' use of another's waste (incidental cross-feeding), much like a beetle feeding on an ungulate's dung. In these cases, cross-feeding is not a cooperative act and poses no challenge for evolutionary theory [7], [8]. However, some instances of cross-feeding may be cooperative, whereby one partner lowers its immediate fitness to benefit another. For example, a species might release a nutrient it would otherwise have used to augment growth of a partner species. These cases pose a challenge to evolutionary theory because the donor species will be selected to avoid releasing the nutrient unless there is some offsetting, greater benefit to itself. Under what circumstances can cross-feeding evolve or be augmented as a system of cooperation?

If any form of between species cooperation is to evolve and be maintained, several criteria must be satisfied [7]–[9]. In particular, the system must be robust to the evolution of exploiters who usurp the benefit provided by others but fail to provide the return benefit. There is now an extensive literature describing the ecologies that render a system resistant to the evolution of cheating [7], [8], and those concepts apply to the evolution of cooperative cross-feeding. The main theme to emerge from this literature reflects Darwin's famous challenge: “Natural selection cannot possibly produce any modification in any one species exclusively for the good of another species” [p. 228: 10]. Thus, cross-feeding must either evolve as a byproduct of one species used by another, hence requiring no ‘modification’ of the donor species, or it must evolve as a reciprocal exchange (reciprocal altruism). Under reciprocity, species A evolves to benefit species B because B in turn benefits A [11].

It is now further appreciated that, for reciprocity to evolve, the reciprocation must benefit the cooperating *individual*, not merely its species. That is, if individual A undergoes a fitness ‘cost’ to help a non-relative, that individual A must personally receive a benefit in return to offset the cost. Furthermore, this return benefit must be above any benefit shared equally by other members of the population to which A belongs. With animals, there are various behavioral mechanisms by which costs and benefits can be directed to individuals. With microbes exchanging resources, the most obvious mechanism of directing resource exchange to individuals is physical proximity, as operates when individuals occupy fixed positions in a structured environment such as a biofilm, or when one species lives inside the other (endosymbiosis). Mixed environments, as when free cells are suspended in liquid, should not encourage the evolution of cooperation via diffusible resources [12].

Beyond this simple understanding, there are several complications. Cross-feeding is not the typical type of cooperation modeled, because the acts are not discrete, and the costs and benefits accrue across generations. Thus, the numbers of individuals are often changing over the course of an extended resource exchange, changing the level of benefits produced and the sharing of those received. This paper offers models to incorporate these dynamical processes into the evolutionary process. As noted above, the literature contains many precedents to suggest that cooperation can evolve only under restrictive conditions. Our focus is specifically on whether and how the dynamical aspects of cross-feeding modify this basic understanding. Our interest is not just in the natural evolution of cross-feeding but also in how cross-feeding might be experimentally evolved for industrial purposes.

## Results

### Full Model

This section offers a model for the growth of two clonal populations interacting by cross-feeding. Let *X* be the local density of type X and let *Y* be the local density of type Y in the same environmental ‘patch,’ to which they are confined. (Roman case is used to designate a type, *italics* to designate density.) The rate of expansion of the X population is affected by three components, its intrinsic ability to grow (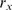), a benefit of cross-feeding from Y, and crowding. Note that 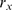 is a growth term, not the relatedness term that is commonly used in models of kin selection. The cross-feeding benefit to X involves the rate constant *b_yx_* (a benefit to X per individual of type Y) times a term that incorporates the numbers of X and Y, to reflect the principle that more cross-feeding resource is provided with larger numbers of Y but the resource must be divided among the X. The per capita level of cross-feeding to an individual of X is thus specified to change as 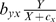. The constant 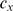 is a damping term that sets the cross-feeding resource proportional to *Y* when *X* is vanishingly small (so that the cross-feeding resource to an individual of X does not approach infinity), but becomes unimportant as *X* grows. Finally, there is logistic growth toward a carrying capacity of *K* combined *X* and *Y* individuals. The same rules apply to *Y*, but with separate parameter values.

The equations for change in *X* and *Y* are thus
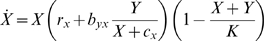
(1.1)

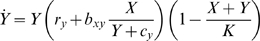
All parameters are non-negative. Allowing positive values for 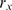 and 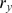 in the absence of cross-feeding means that each species can grow in the absence of the other. Cross-feeding merely augments that growth.

These equations are strictly dynamical. To accommodate adaptive evolution among genotypes with different parameter values, we suppose that different X, Y genotype pairs are each growing in separate local patches. After a set time for growth, individuals from different patches are mixed and redistributed at low density into separate patches to start the dynamical process again. Genotypes that achieve the highest local densities within a patch would then increase their proportions in subsequent rounds of mixing and growth. At this stage of the model, different genotypes of X (and of Y) are not allowed to compete in the same patch (modified below). This process can certainly be applied in an experimental context of artificial selection, though not necessarily mimicking any natural process. However, its basic structure is similar to that of Maynard Smith's haystack model of group selection [13] for the evolution of cooperation within a species. Some key differences from that haystack model are (i) interactions occur between two species; (ii) each patch is constituted with a single individual or genotype of each species; (iii) we will specifically vary the time at which mixing occurs among patches, rather than allow the patch dynamics to reach an equilibrium.

The essence of cross-feeding evolution as a form of cooperation is that an individual is favored to increase its cross-feeding contribution to the other type above the level that would evolve in the absence of the other type. Within the framework of this model, this question is interesting and biologically relevant only if a trade-off exists between 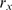 and 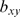 (and between 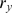 and 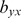). Such a trade-off means that an individual sacrifices its own intrinsic growth rate to facilitate growth of another species, so any level of cross-feeding to a partner involves immediate sacrifice. The models can be used to identify which combinations of 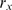 and 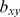 (and of 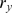 and 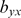) are favored given this trade-off.

The evolutionary consequences of eqn (*1.1*) within this biological setting are sensitive to the dynamics of population growth. Specifically, the duration of growth allowed between mixing affects selection. There are three density phases with different outcomes, and these will be considered separately below.

### Growth at Low Density

When *X* and *Y* are small relative to the *c* and *K*, the system obeys approximately
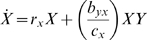
(1.2)

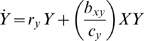
Equations (*1.2*) represent a form of Eigen's hypercycle [14], with *X* and *Y* growing faster than exponentially as the *XY* terms dominate the equations. However, these equations only apply here for the lowest of densities, at which this greater-than-exponential growth does not operate. It is easy to appreciate that, as long as densities remain very low, cross-feeding will not be favored because the contribution to growth from cross-feeding is too small. That is, when *X* and *Y* are both small, their product will be even smaller, too small for an increase in *b* to offset any decrement to *r*. The equations are then dominated by the 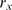 and 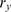 terms:
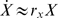
(1.3)

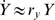
Biologically, this conclusion reflects the intuitive point that low densities do not provide enough absolute cross-feeding resource to justify sacrificing intrinsic growth.

The model depicts local densities of *X* and *Y* in a patch. Since the patch must include at least one individual of each type to be considered by these equations, (*1.3*) will not apply to the minimal density in some empirical conditions. Thus, in an experimental setting, low density can possibly be avoided by clustering the two individuals that colonize a patch while keeping the density of patches low. However, if low initial densities cannot be avoided, it may suffice to allow growth to continue into the realm of intermediate density, in which cross-feeding can be favored, as considered next.

### Growth at Intermediate Density

When *X* and *Y* are large relative to the 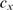 and 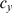 but their sum is still moderately small relative to the carrying capacity *K*, the system tends toward
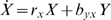
(1.4)

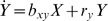
This set of differential equations is linear and is easily solved. The two populations ultimately grow according to 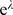, where 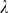 is the largest eigenvalue associated with the transition matrix in (*1.4*):
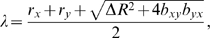
(1.5)where 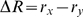. Understanding how the parameter values affect 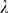 gives insight to selection of cross-feeding at intermediate densities. Note that, as long as 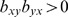, both populations *X* and *Y* will grow at rate 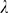. (That is, the eigenvector associated with 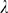 has positive entries for both *X* and *Y*.) Thus, during this stage of growth, natural selection will favor increases in 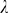 even though one population may be larger than the other throughout this growth.

Some useful properties of this growth rate equation can be noted by inspection. First, the cross-feeding terms 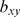 and 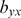 enter only as a cross product. If either is zero, then cross-feeding disappears as a contribution to growth of the pair. It can thus be inferred that the impact of cross-feeding on growth rate is limited by the smaller *b*. Second, growth rate appears to improve with the magnitude of the difference between 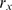 and 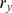. Thus, if the sum 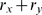 is held constant, increasing their difference (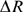) improves fitness. Some insight to this strange result can be understood from the fact that, when 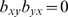 (i.e., when bidirectional cross-feeding ceases), the largest eigenvalue is merely the larger of 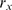 and 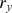. With bidirectional cross-feeding, inclusion of 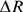 in the equation for 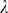 - the largest eigenvalue - accounts for the fact that 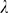 must be at least as large as the larger of 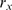 and 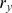, not their average (their average appears in front of the radical in equation *1.5*).

Our interest is in how selection will act on variation in the parameters controlled by X or Y (e.g., 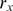 and 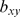). This understanding is obtained from the derivative of 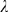 with respect to 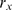, treating 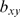 as a decreasing function of 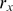 to reflect the trade-off. If 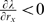, selection favors decreases in 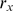, and by virtue of the trade-off, will favor the coupled increases in 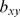. Thus 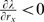 indicates selection for increased cross-feeding. These conditions are met when
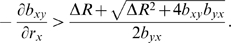
(1.6)The left side of this inequality is strictly positive, since the trade-off requires 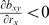.

It is easiest to comprehend selection for increased cross-feeding at the boundary 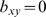, when X initially provides no cross-feeding to Y. At this point in the parameter space, condition (*1.6*) becomes
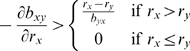
(1.7)Several new implications are now evident. One is that when 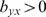 (when Y initially provides a benefit), selection always favors reciprocal cross-feeding from X to Y when 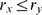 (when the intrinsic growth rate of Y is already the larger one). This unintuitive result derives from the fact that 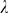 is always at least as large as the larger of 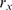 and 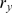. When 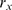 is the smaller of the two, reducing it further to enhance cross-feeding is more than offset by the feedback through the coupled growth rate of Y.

A second implication is that trade-offs with big gains in 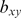 per decline in 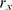 enhance the evolution of cooperation. This conclusion follows because those trade-offs yield large values on the left-hand side of (*1.6*) and increase the parameter range permitting the evolution of cross-feeding. The relevance of this basic principle for reciprocal altruism was emphasized several decades ago and again recently [9], [15] and is easily appreciated intuitively–a small, up-front cost that feeds a large benefit to the partner in a reciprocal relationship needs to pay off only modestly per benefit to the partner. Third, large values of cross-feeding from Y to X (large 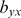) enhance the evolution of reciprocity in the other direction, from X to Y. This follows from the fact that 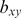 and 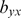 enter as a product, so that large values of 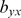 translate into large returns when X invests in Y. Conversely, if Y provides no cross-feeding benefit to X, then X cannot be selected to help Y [9]. This suggests that cross-feeding as cooperation must initially evolve from a system in which at least one of the directions of cross-feeding is maintained as an incidental byproduct, not involving a cost to the donor (not cooperative).

### Growth at High Density

For the full model in (*1.1*), the equilibrium densities of *X* and *Y* depend on initial conditions, so there is no unique solution except that their sum, *X+Y*, equal the carrying capacity, *K*. Nonetheless, some qualitative outcomes can be identified. Importantly, in the absence of cross-feeding, the type with the highest intrinsic growth rate (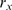 or 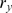) will always reach the higher density if initial densities are equal. If 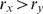, for example, the final density of X can vastly exceed the final density of Y. Introduction of bidirectional cross-feeding will then usually lower the final density of *X*, because the effect of cross-feeding is to raise the densities of both *X* and *Y* together, ultimately preventing either from greatly outpacing the other. Consequently, even when cross-feeding is beneficial to both types at intermediate densities, it will usually be detrimental to one when growth is allowed to continue to high densities.

This high density effect arises because cross-feeding does not augment the resource that limits total density - the benefit of cross-feeding does not affect the carrying capacity, only the time to get there. Thus, enhancing the growth rate of one's partner may feed back in the short term to enhance growth rates of both X and Y, but the partner eventually becomes a competitor when the common resource becomes limiting.

#### From low to high density

**Figure 1:**
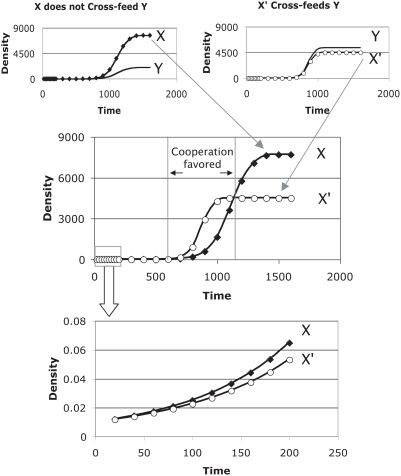
Simulations of two-species populations reveal the three phases of selection (based on equations *1.1*). The top level shows the dynamical trajectories of isolated populations of two (X,Y) genotype pairs differing in the level of cross-feeding provided by the X genotype; X does not cross-feed but X' does cross-feed to Y. Y cross feeds to X at the same level in both pairs, so the parameters of Y are the same in both simulations. The X and X' types are both represented by the curves marked by symbols (filled squares for *X*, open circles for *X'*), whereas the curves for type Y have no symbols (top level). The middle panel compares in the same graph the densities achieved by X and X', revealing that the cross-feeding X' outgrows X only at intermediate densities; the zone in which *X'* exceeds *X* is indicated by the vertical bars. The lower panel shows on an expanded vertical scale that X outgrows X' at low densities despite its later disadvantage. Densities of X and Y were both started at 0.01, with 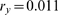 and 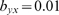. In the simulation illustrated on the left 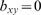 and 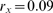. On the right 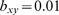 and 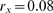. Carrying capacity (*K*) was set at 10,000.

**Figure 2:**
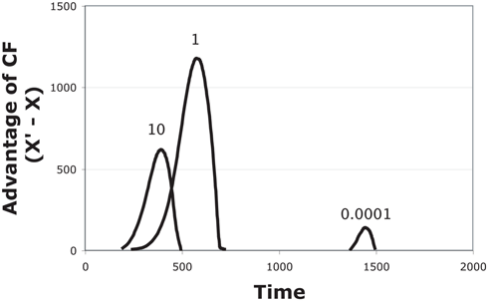
The advantage of cross-feeding changes with initial densities of the bacteria (based on simulations of (*1.1*)). As in fig. 1, the cross-feeding X' genotype outgrows the non-cross-feeding X at intermediate densities. However, the times at which X' exceeds X and the magnitude of the excess depend on starting density. Curves are labeled according to the starting densities, the same for all genotypes, X, X' and Y, within a trial. The advantage of X' is diminished at high and low initial densities. In contrast to fig. 1, the curves here depict only the excess of X' over X during a run (showing *X'*–*X*, where ever that value exceeds zero). The curve for an initial density of 0.0001 reveals a slight advantage of cross-feeding for only 100 time units. The curve for an initial density of 1 reveals both the largest advantage of cross-feeding and the longest benefit (425 time units). The curve for an initial density of 10 reveals a modest advantage of cross-feeding spanning 275 time units. Parameters for Y were r_y_ = 0.011, and b_yx_ = 0.01; for X' were r_x_ = 0, and b_xy_ = 0.01; for X were r_x_ = 0.008, and b_xy_ = 0. K = 10,000 for all runs.

By comparing different genotypes across patches, simulations reveal all three phases in a single trajectory (Fig. 1 illustrates one of many examples). At low density, a cross-feeding genotype does worse than a non-cross-feeding genotype because the reduction in intrinsic growth rate cannot be offset by the small gains from cross-feeding, matching our conclusions based on the approximate equations (*1.3*) (Fig. 1, bottom). At intermediate densities, before carrying capacity has much impact on growth rates, a cross-feeding genotype can outgrow a non-cross-feeding genotype because of the synergistic feedback it receives (Fig. 1, middle). Finally, as high density is approached, one type will generally be held back by cross-feeding. If growth is continued to high density, all benefits of cross-feeding will be erased for one of the pair (Fig. 1, middle). The advantage of cross-feeding during growth at intermediate densities also depends on the period of growth from low initial frequencies, so the populations must be started at appropriate densities to observe an appreciable benefit (Fig. 2). Simulations further support the unintuitive dichotomous behavior of cross-feeding advantage indicated by eqn (*1.7*) (data not shown).

### Exploitation

To here, the models have been argued to apply to local populations (patches), such as would operate between colonies, each with a pair of bacterial strains growing in isolation of other colonies. At some point, the populations will expand and become large enough that other types migrate in or other types arise by mutation. These larger populations will be vulnerable to exploiting genotypes (often referred to as ‘cheaters’) who share in the cross-feeding resources from the other type but do not reciprocate in cross-feeding themselves. By virtue of the trade-off between *r* and *b*, these exploiters will enjoy a higher intrinsic growth rate than their counterparts who do cross-feed. A set of equations corresponding to (*1.1*) but including an equation for exploiters derived from type X (X_e_) is
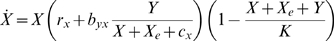
(1.8)

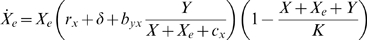


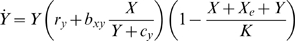
where 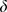 is the gain in intrinsic growth rate of type X_e_ from abandoning cross-feeding to Y.

Thus, exploiters X_e_ enjoy the same level of benefits from Y as do the cross-feeding X, but they also have a higher intrinsic growth rate than X because they do not sacrifice to cross-feed Y. As a consequence, the exploiting X_e_ will outgrow the cooperating X, and the level of cross-feeding provided by X will fall. Ultimately, Y will be selected to abandon investing any cooperative cross-feeding to X, and the system will return to initial levels determined by the non-cooperative components of cross-feeding.

### Implications for Artificial Selection

As one of our motivations is to understand how artificial selection might favor cross-feeding in a laboratory setting, this section considers how the foregoing models guide the design of those experimental protocols. We suppose that the organisms are microbes such as bacteria. A foremost requirement for the selection of enhanced bidirectional cross-feeding is that cross-feeding already exist in at least one direction, say from *X* to *Y*. In this way, mutants of *Y* that reciprocate will automatically receive feedback from enhancing their partners. Beyond this observation, the models highlight two broad issues in selecting and maintaining cross-feeding as a form of cooperation: selection is sensitive to the dynamical stages of population growth, and some ecologies allow the invasion of exploiting genotypes that work against the evolution of cross-feeding.

There is already a literature dealing with the second of these: how to avoid exploitation in the evolution of cooperation. The main message from that prior work is that some form of group structure is required so that resource exchange happens locally [7], [8], [12]. Indeed, the haystack model (on which our model is based) is one of group selection. Thus individuals and their immediate descendants that provide a benefit to another species or strain personally receive the reciprocation for providing that benefit because they exist in groups to the exclusion of other genotypes. The ideal design is thus to establish pairs of individuals (one of each species), with each pair grown–producing descendants–in physical isolation from other pairs, ultimately resulting in isolated colonies of different genotype pairs. For many types of microbes, colony growth retains spatial proximity of descendants during growth. The more cooperative pairs may be evident as larger colonies. If genotypes with enhanced cooperative properties cannot be individually identified during this growth, the entire population of colonies can be mixed and re-established as pairs, and the process repeated indefinitely to achieve long term selection of enhanced cross-feeding. In practice, it may be difficult to invariably establish paired individuals of different genotypes, so this design may be approximated by distributing individuals at low densities on plates for subsequent growth into colonies. It may also be necessary to supplement the media with enough of the limiting resource to enable sufficient growth to surpass low density thresholds that inhibit selection of cross-feeding, or to use a higher density of the species that already cross-feeds.

The dynamical constraints on the successful selection of cross-feeding pose a different challenge. We can use the models to identify the problem, but there is no universal protocol to avoid inappropriate densities as there is for avoiding exploitation. In essence, the solution to optimizing selection over appropriate densities is merely to avoid growth at densities that are too high or too low. These conditions will depend on the specifics of each system, including the production rates and diffusion constants of the resources that underlie cross-feeding. A simple, empirical way to avoid densities that are ‘too high’ may be simply to avoid growth to the point that most of the population has reached saturation.

## Discussion

Reciprocal cross-feeding among microbes represents a type of mutualism [16]. It may have two fundamentally different evolutionary origins, however, one an incidental exchange of waste products from one species that benefits another, or instead as a cooperative act by one species evolved specifically to enhance growth and survival of another. This study has addressed the latter: what conditions favor the evolution of cross-feeding as a cooperative act? Since Hamilton [17], [18], the standard models for evolution of cooperation have considered the exchange of discrete fitness acts between pairs of individuals [as in payoff matrices, 19]. The evolution of cooperative cross-feeding does not trivially lend itself to that approach because of dynamics: the exchanges are quantitative traits supplied continuously to populations, and the numbers of individuals in those populations are changing during the exchanges and because of the exchanges.

The model developed here is strictly dynamical, describing the growth of two interacting populations (species), but it enables the inference of evolution by a couple of devices. First, the model accommodates natural selection in a spatial context, by supposing that different genotypes compete in separate patches, isolated from each other. Growth continues for a while, at which point the individuals from different patches are mixed and settled at low density into new patches. Over many cycles, genotypes that grow to the highest densities within patches will dominate the population. The characteristics favored by selection in this type of process are found as the parameter values maximizing a genotype's growth up to the time that the populations in different patches are mixed. To accommodate evolution within populations, additional equations are added.

The model revealed several factors enhancing the evolution of cooperative cross-feeding. These may be conveniently partitioned into general evolutionary factors and dynamical factors. General factors are those that have been identified in the more classic models for the evolution of cooperation (noted below), whereas dynamical factors are those specific to the cross-feeding context.

Three types of general factors were observed to affect the evolution of cooperative cross-feeding. Recognition of these factors as being important is not new to our study, but the fact that they were observed to be important here as well as in prior work strengthens confidence in the model:

### 1. Population structure

The return benefits of providing cooperation must be directed to individuals or clones (colonies), not to entire populations. This point has been recognized broadly for the evolution of cooperation [7], [8], [9], [12], and with microbes, is usually interpreted as a requirement for spatial structure. Our model invoked strong spatial structure for the growth phages of the populations. However, in our model, mixing is important at one step of the life cycle, after the growth phase, so the structure should not be maintained indefinitely (as pointed out by a reviewer).

### 2. Initial conditions

Cross-feeding must pre-exist in one direction for it to evolve cooperatively in the other direction. Thus, cooperative cross-feeding is likely to evolve only if cross-feeding in one direction is incidental, not cooperative. Reciprocation is an essential component to all models for the evolution of cooperation between species [9].

### 3. Fitness effects

Cross-feeding is more easily selected when its cost to the donor is low per benefit to the recipient and when the recipient already provides a large cross-feeding benefit to the donor. The feedback loop is enhanced by both effects. The former point has been concluded from other models of reciprocal altruism [9], [15].

The novelty of this study is to address the role of population dynamics in the evolution of cooperative cross-feeding. The main dynamical result is that, even when the above properties apply, cooperative cross-feeding is favored only during growth at intermediate population densities. At low density, the return benefit is too slight to offset the per-individual cost of providing a cross-feeding resource. This hurdle can sometimes be overcome by allowing populations started at low density to grow up to intermediate density, but the low-density growth may also overwhelm the benefits of cooperation at intermediate density. At high density, the partner species becomes a competitor for resources needed by both species. Given our assumption that each species can grow by itself at least slowly, each species could reach high density on its own, so the faster-growing species is ultimately held back by facilitating growth of the slower species. This high density result has a broad parallel in kin selection theory: cooperative acts are favored among close relatives except when kin are each other's closest competitors [20], [21]. Paradoxically, while spatial structure is essential for the evolution of cooperative cross-feeding at intermediate densities, it is also responsible for the selection against cross-feeding at high density; spatial structure more generally underlies the evolution of diffusible antagonistic interactions among competitors [22].

The conclusions derived from the models here are supported by an experimental study in which cooperative cross-feeding was evolved in a *Salmonella enterica* Serovar *typhimurium* to aid an *Escherichia coli* unable to synthesize methionine when the pair was grown in lactose minimal media (Harcombe, unpublished). In the presence of methionine, the *E. coli* could metabolize lactose, but the *Salmonella* could not. Metabolism of lactose by *E. coli* resulted in excretion of a carbon source (likely acetate) that enabled the *Salmonella* to grow. Thus, if the *Salmonella* excreted enough methionine, the system would be maintained through reciprocal cross-feeding. The initial strain of *Salmonella* did not secrete enough methionine to maintain the system, but joint propagation of both species in lactose minimal media on plates rapidly led to a *Salmonella* mutant that overproduced methionine at a sufficient level to maintain both species. This mutant was identified by large colony size (consisting of both species), but only after several days of growth that allowed the mutant colony to outgrow non-mutant colonies.

This bacterial system thus exhibits several properties identified by our model as promoting the evolution of cooperative cross-feeding: spatial structure, an initial one-way cross-feeding that was not cooperative, and growth to intermediate densities. The quantitative fitness consequences of the cost to cross-feeding by *Salmonella* and the benefit provided by *E. coli* were not measured.

It is widely appreciated that cooperation can evolve only under restrictive ecologies, and much of the attention to this problem has been directed at testing whether natural systems of cooperation meet those ecologies [9]. The results here potentially add a new layer of challenge to the evolution of cooperation, not only for cross-feeding, but potentially to other mutli-species systems. Yet it remains to be shown just how restrictive these dynamical constraints may be. Literal application of our model would seem to render the evolution of cooperative cross-feeding nearly insurmountable in natural settings–and lead to the conclusion that probably all natural cross-feeding is incidental–but there are reasons against accepting this conclusion without further study. Specifically, the dynamical constraints identified here may be compatible with a much wider range of ecologies and bacterial behaviors than assumed in our model. For example, bacteria may be able to respond conditionally to the abundance of other bacteria (e.g., through quorum sensing), enabling cooperative behavior to be turned on and off at appropriate times [e.g., 23]. In this case, cooperative cross-feeding could operate at appropriate densities without requiring that the bacteria never experience inappropriate densities (as assumed by our model). The life styles of bacteria in biofilms, involving cycles of growth in a structured environment followed by dispersal, may also be broadly compatible with our model. Regardless of the difficulty of evolution of cooperative cross-feeding in natural settings, however, the models guide the design of methods to achieve it through artificial selection.

## Analysis

Analytical results were derived manually. Numerical iterations used C++ code of the equations for the full model (1.1), compiled in CodeWarrior™, calculated at intervals of *dt* = 0.0001 time units. Parameter values of the numerical trials shown in the figures are provided in the legends.

## References

[1] Pernthaler A, Dekas AE, Brown CT, Goffredi SK, Embaye T (2008). Diverse syntrophic partnerships from deep-sea methane vents
revealed by direct cell capture and metagenomics.. Proc Natl Acad Sci U S A.

[2] Schink B (2002). Synergistic interactions in the microbial world.. Antonie Van Leeuwenhoek.

[3] Schink B, Ballows AT, H G, Dworkin M, Schleifer KH (1991). Syntrophism among prokaryotes.. The prokaryotes. 2nd ed.

[4] Hallam SJ, Putnam N, Preston CM, Detter JC, Rokhsar D (2004). Reverse methanogenesis: testing the hypothesis with environmental
genomics.. Science.

[5] Overmann J, Schubert K (2002). Phototrophic consortia: model systems for symbiotic
interrelations between prokaryotes.. Arch Microbiol.

[6] Rawlings DE, Johnson DB (2007). The microbiology of biomining: development and optimization of
mineral-oxidizing microbial consortia.. Microbiology.

[7] Sachs JL, Mueller UG, Wilcox TP, Bull JJ (2004). The evolution of cooperation.. Q Rev Biol.

[8] West SA, Griffin AS, Gardner A, Diggle SP (2006). Social evolution theory for microorganisms.. Nat Rev Microbiol.

[9] Foster KR, Wenseleers T (2006). A general model for the evolution of mutualisms.. J Evol Biol.

[10] Darwin C (1859). The Origin of Species.

[11] Trivers RL (1971). The evolution of reciprocal altruism.. Quarterly Review of Biology.

[12] West SA, Buckling A (2003). Cooperation, virulence and siderophore production in bacterial
parasites.. Proc Biol Sci.

[13] Maynard Smith J (1964). Group selection and kin selection.. Nature.

[14] Eigen M, Schuster P (1979). The Hypercycle: A principle of natural self-organization.

[15] Schaffer WM (1978). A note on the theory of reciprocal altruism.. The American Naturalist.

[16] West SA, Griffin AS, Gardner A (2007). Social semantics: altruism, cooperation, mutualism, strong
reciprocity and group selection.. J Evol Biol.

[17] Hamilton WD (1964). The genetical evolution of social behavior 1.. Journal of Theoretical Biology.

[18] Hamilton WD (1964). The genetical evolution of social behavior 2.. Journal of Theoretical Biology.

[19] Maynard Smith J (1982). Evolution and the Theory of Games.

[20] West SA, Pen I, Griffin AS (2002). Cooperation and competition between relatives.. Science.

[21] Taylor PD (1992). Altruism in viscous populations - an inclusive fitness approach.. Evolutionary Ecology.

[22] Chao L, Levin BR (1981). Structured habitats and the evolution of anticompetitor toxins in
bacteria.. Proc Natl Acad Sci U S A.

[23] Nadell CD, Xavier JB, Levin SA, Foster KR (2008). The evolution of quorum sensing in bacterial biofilms.. PLoS Biol.

